# Commentary: Oil and gas development exposure and atrial fibrillation exacerbation: a retrospective study of atrial fibrillation exacerbation using Colorado's all payer claims dataset

**DOI:** 10.3389/fepid.2025.1480372

**Published:** 2025-02-10

**Authors:** Judy Wendt Hess

**Affiliations:** Hess Epidemiology Services LLC, Houston, TX, United States

**Keywords:** atrial fibrillation, survival analysis, interrupted time series, oil and gas development, Colorado (USA)

A Commentary on Oil and gas development exposure and atrial fibrillation exacerbation: a retrospective study of atrial fibrillation exacerbation using Colorado’s all payer claims dataset By McKenzie LM, Allshouse WB, Abrahams B and Tompkins C (2024). Front. Epidemiol. 4:1379271. doi: 10.3389/fepid.2024.1379271

## Introduction

McKenzie et al. (1) recently evaluated whether individuals with pre-existing atrial fibrillation (AF) or atrial flutter (AFl) living within 1 mile of an oil and gas (O&G) well were more likely to experience a healthcare encounter for AF/AFl during or after, compared to before, well development. The analysis used 2009–2017 data from Colorado's All Payer Claims Database (APCD) (2), representing nearly two-thirds of the state's insured residents.

The study was described as a quasi-experimental interrupted time series (ITS); however, it did not satisfy ITS design characteristics or statistical requirements, and to describe it as such ascribes rigor to the analysis that the structure of the data was unable to provide. The paper further detailed the use of survival analysis (SA), but again, the analysis did not include critical elements of this statistical approach.

Readers who have less familiarity with these statistical techniques may take these study results at face value, without understanding that the authors applied what are perceived as rigorous methods to data that were not suited for them. The methodological issues with this analysis, discussed below, should be addressed by the authors before the study is accepted as epidemiological evidence that nearby O&G activity increases the risk of AF/AFl.

## Interrupted time series

ITS is the real-world analog to a randomized controlled trial (RCT) (3). As described in papers cited by McKenzie et al., ITS evaluates the impact of an intervention introduced to a specific population at a clearly defined point in time, effectively controlling for between-group differences and underlying time trends in the outcome (4, 5). The objective is to describe whether increased disease occurrence coincided with the start of the intervention (6). Results are typically reported as the average number or rate of health events before and after the intervention (7).

The authors chose the ITS design because it allowed for control of potential confounders unavailable in the APCD. However, this feature of ITS hinges on the intervention beginning and ending at approximately the same time across the study population, similar to RCT participants. The intervention in this study was the development of a well within 1 mile of a home address, starting with the spud date and ending on the first day of production. This “during” drilling (i.e., intervention) period was highly variable across study participants, ranging from 3 to 844 days. Periods “before” and “after” the intervention were the same duration as the “during” period, but one before the spud date and the other after the first production date. Thus, follow-up periods including before, during, and after drilling for the nearly 1,200 study participants were as short as 9 days for some and over 2,500 days for others, staggered across the 9-year study period. While statistical approaches to analyze multiple baseline time series data exist, they cannot accommodate both staggered intervention start dates and widely varying follow-up time across the study population (8).

## Survival analysis

The authors do not explain why multi-failure SA was used, which is not a typical or recommended statistical method for ITS (9, 10). The study reported hazard ratios—the estimate of risk produced by SA—comparing the risk of AF/AFl encounters during vs. before drilling and after vs. before drilling.

SA requires calculating the observed person-time for each participant (11) based on the period of time he or she was “at risk” of a recorded AF/AFl encounter, which presumes presence in the APCD with no gaps in coverage. Person-time could almost certainly have been derived from beneficiary enrollment dates in the APCD; however, no description of this was included in the paper. Rather, it appears that at-risk and control patients were assumed to be present in the APCD for the entirety of their follow-up periods.

More concerning was the exclusion of half the at-risk patients from the analysis “to reduce errors from unknown losses to follow-up.” McKenzie et al. describe these participants in their text as “without a claim of any type preceding the before period and succeeding the after period,” and alternatively in their Figure 2 as “without evidence of presence in APCD through follow-up” (1). However, since an AF/AFl diagnosis between 2009 and 2017 (but before the end of follow-up) was required for study eligibility, and the same APCD enrollment file from which the authors obtained address, gender, and birth date also included enrollment dates, the reason for excluding these patients is unclear. Excluding those with no claims during follow-up from the at-risk population would effectively inflate hazard ratios by removing person-time from the denominator that did not add any AF/AFl events to the numerator.

Finally, the lack of covariate data in the APCD, while not required for ITS, is required for SA, particularly when participant follow-up time is variable and not temporally aligned. Although the study included controls, this could not account for factors that varied with time because the controls were matched to at-risk patients not by calendar year, but rather by region of residence in the state and year of first AF/AFl diagnosis in the APCD. In addition to seasonal trends and increasing AF prevalence during the 9-year study period (12), technological, regulatory, and economic factors likely impacted underlying trends in noise and air pollution exposure resulting from nearby drilling over time.

## Conclusion

The question addressed in the study by McKenzie et al.—whether close proximity drilling exacerbates existing AF/AFl, and if so, whether certain demographic groups are particularly susceptible—is an important one that has not been previously addressed. The design of the study was novel, and the dataset used in their analysis is a rich source of claims data from a state with considerable O&G activity.

The authors state in their conclusion: “these findings support development of mitigation strategies and regulations to protect the health of populations living near O&G well sites.” The methodological issues described above, however, are significant and raise concerns about the validity and interpretation of the reported results. Given the importance of this topic, and potential implications for public health practitioners and policymakers, I encourage the authors to address these methodological issues in any further research they conduct on this topic.
